# Supplementary material for: A Study on the Static Magnetic and Electromagnetic Properties of Silica-Coated Carbonyl Iron Powder after Heat Treatment for Improving Thermal Stability
Source: Materials (Basel). 2022 Mar 28;15(7):2499. doi: 10.3390/ma15072499 (PMC8999542; doi:10.3390/ma15072499)
Supplement: Supplementary file 1 [file materials-15-02499-s001.zip › materials-1615251-supplementary.pdf]

materials

MDPI

Article

A Study on the Static Magnetic and Electromagnetic Properties of Silica-Coated Carbonyl Iron Powder after Heat Treatment for Improving Thermal Stability

Xu Yan, Xinyuan Mu, Qinsheng Zhang, Zhanwei Ma, Chengli Song * and Bin Hu *

State Key Laboratory for Oxo Synthesis and Selective Oxidation, Lanzhou Institute of Chemical Physics, Chinese Academy of Sciences, Lanzhou 73000, China; yanxu@licp.cas.cn (X.Y.); muxinyuan@licp.cas.cn (X.M.); zhangqinsheng@licp.cas.cn (Q.Z.); mazhanwei@licp.cas.cn (Z.M.)

* Correspondence: songchl@licp.cas.cn (C.S.); hcom@licp.cas.cn (B.H.)

Abstract: In order to study the thermal stability of coated carbonyl iron powder (CIP) and its influence on magnetic properties, carbonyl iron powder was coated with a silica layer and then annealed in an air atmosphere at elevated temperatures. Transmission electron microscopy (TEM) analysis and Fourier transform infrared spectroscopy confirmed the existence of a silicon dioxide layer with a thickness of approximately 80~100 nm. Compared with uncoated CIP, the silicon-coated CIP still maintained a higher absorption performance after annealing, and the calculated impedance matching value Z only slightly decreased. It is worth noting that when the annealing temperature reached 300 °C, coercivity (H_c) increased, and the real and imaginary parts of the permeability decreased, which means that the silicon dioxide layer began to lose its effectiveness. On the contrary, the significant decrease in microwave absorption ability and impedance matching value Z of uncoated CIP after annealing were mainly because the newly formed oxide on the interface became the active polarization center, leading to an abnormal increase in permittivity. In terms of the incremental mass ratio after annealing, 2% was a tipping point for permeability reduction.

Keywords: microwave absorption; thermal stability; silica coating; carbonyl iron powders

Citation: Yan, X.; Mu, X.; Zhang, Q.; Ma, Z.; Song, C.; Hu, B. A Study on the Static Magnetic and Electromagnetic Properties of Silica Coated Carbonyl Iron Powder after Heat Treatment for Improving Thermal Stability. *Materials* **2022**, *15*, 2499. https://doi.org/10.3390/ma15072499

Academic Editor: Anton Nikiforov

Received: 12 February 2022
Accepted: 21 March 2022
Published: 28 March 2022

Publisher's Note: MDPI stays neutral with regard to jurisdictional claims in published maps and institutional affiliations.

Copyright: © 2022 by the authors. Submitted for possible open access publication under the terms and conditions of the Creative Commons Attribution (CC BY) license (https://creativecommons.org/licenses/by/4.0/).

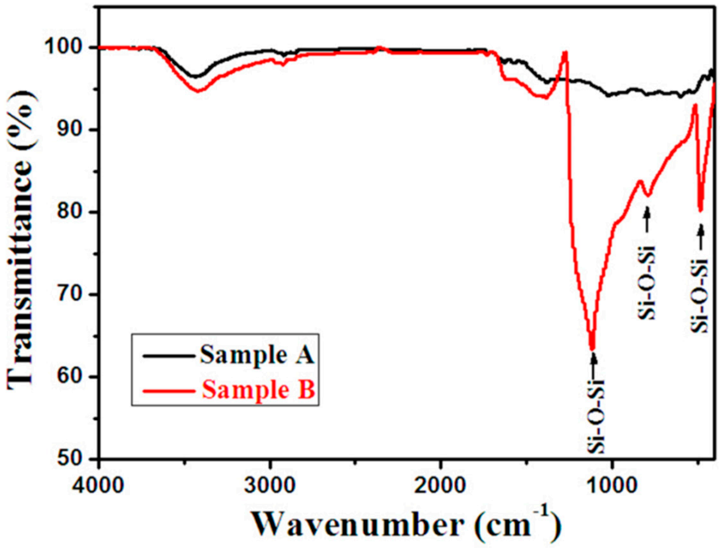

Figure S1. Fourier transform infrared spectroscopy (FT-IR) spectra of Sample A and Sample B.

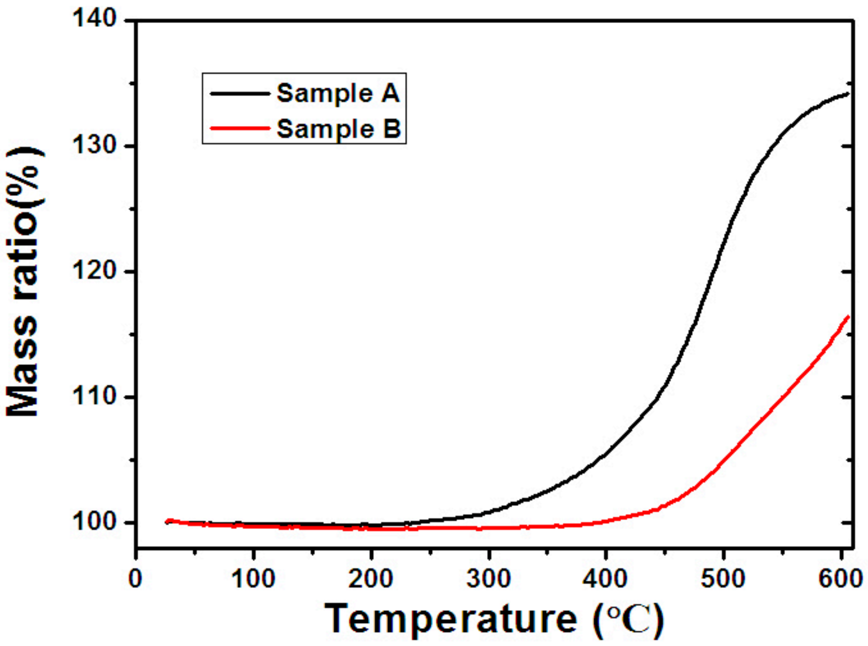

Figure S2. The thermogravimetric (TG) curves of Sample A and Sample B in air atmosphere.

Figure. S1 shows the FT-IR spectra of Sample A and Sample B. It can be observed that there are characteristic SiO_2 peaks in Sample B, the peak at 1118 cm^{-1} is ascribed to the asymmetric stretching of Si–O–Si bond of silica; the peak at 787 cm^{-1} originates from symmetric stretching of Si–O–Si bond.

Figure. S2 shows the TG curves of Sample A and Sample B from 25 °C to 600 °C in an air atmosphere with a 10 K per minute velocity. The mass ratio of Sample A remained constant before 250 °C, then increased significantly due to oxidation, and gradually slowed down at 600 °C, indicating the end of the oxidation process. At 600 °C, the maximum mass ratio is 134%, corresponding to the formation of Fe_3O_4, which can be confirmed by the XRD pattern (Figure 2a). However, the mass ratio of Sample B remained unchanged before 400 °C and then rapidly increased with elevated temperature. Thus, we can observe the violent oxidation process of Sample B is significantly delayed, indicating the SiO_2 layer effectively prevents the oxidation of the internal iron powder.

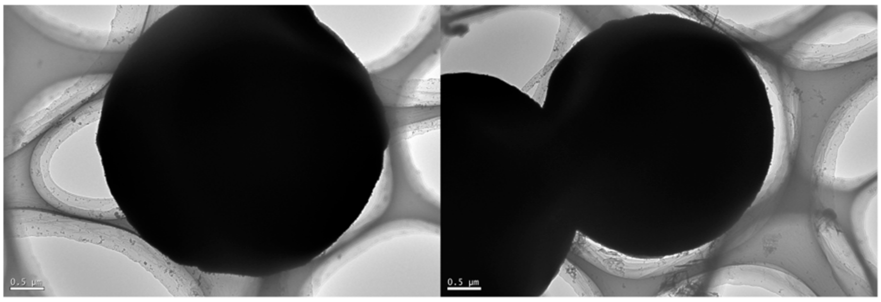

Figure S3. The Transmission Electron Microscope (TEM) photos of Sample B annealing at 300 °C.

Figure. S3 shows the TEM photos of Sample B annealing at 300 °C. Sillica coating is not observed on the particles surface.

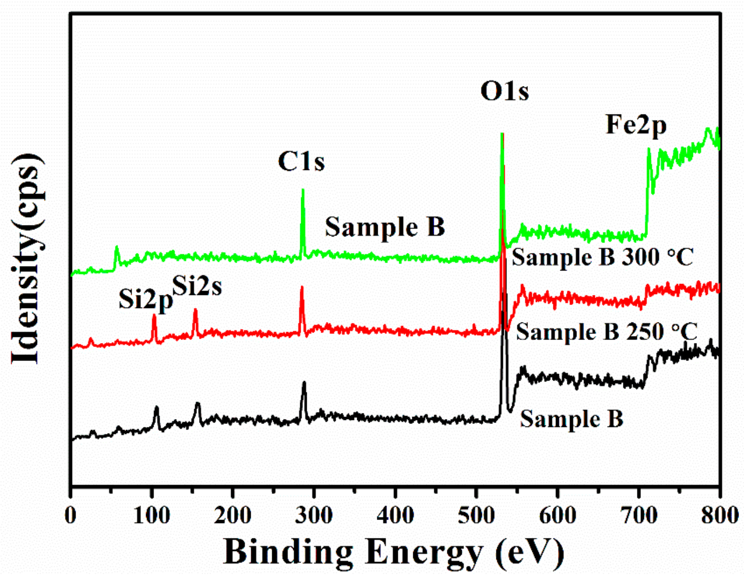

Figure S4. The X-ray photoelectron spectroscopy (XPS) spectra of Sample B, Sample B annealing at 250 °C, and Sample B annealing at 300 °C.

Figure. S4 shows the XPS spectra of Sample B, Sample B annealing at 250 °C, and Sample B annealing at 300 °C from 0 to 800 eV. It is observed that there are no Si2p and Si2s peaks in the spectrum of Sample B annealing at 300 °C. The result is consistent with that of sFig. 3, indicating the silica coating of Sample B might peel off from the particles surface after annealing at 300 °C.
